# Do Antinuclear Antibodies Influence the Clinical Features of Chronic Spontaneous Urticaria?: A Retrospective Cohort Study

**DOI:** 10.1155/2022/7468453

**Published:** 2022-11-29

**Authors:** Sittiroj Arunkajohnsak, Sukhum Jiamton, Papapit Tuchinda, Leena Chularojanamontri, Chuda Rujitharanawong, Nattacha Chanchaemsri, Kanokvalai Kulthanan

**Affiliations:** Department of Dermatology, Faculty of Medicine Siriraj Hospital, Mahidol University, Bangkok 10700, Thailand

## Abstract

**Background:**

Antinuclear antibody (ANA) is often used as a screening test for autoimmune comorbidities in patients with chronic spontaneous urticaria (CSU). However, the relationship of ANA status and the clinical course of the disease have not been fully described.

**Objectives:**

To compare clinical features of CSU patients who are positive and negative for ANA.

**Methods:**

This was a retrospective cohort study that enrolled CSU patients attending the Urticaria Clinic at Siriraj Hospital from 2013 to 2019. Demographics, clinical characteristics, laboratory investigations, and treatments were collected until July 2021. All patients were investigated for ANA. Clinical feature data was compared between CSU patients with positive ANA and negative ANA groups using the 2-sample *t*-test and the Mann–Whitney *U* test for quantitative variables. The chi-squared test or Fisher's exact test was conducted to explore the association of qualitative variables. Disease relapse and remission were analysed via Kaplan-Meier survival analysis.

**Results:**

Of 323 CSU patients, 31% had positive ANA. There were no significant differences in disease severity or impairment in quality of life. Patients with a positive ANA test had significantly lower prevalence of allergic rhinitis (*p* = .048) and significantly higher level of erythrocyte sedimentation rate (*p* = 0.007). Kaplan-Meier survival analysis showed that 2% of ANA positive CSU patients achieved remission status after one year and 28% did so after five years. There was no statistically significant difference in time to remission and time to relapse between ANA-positive and negative groups.

**Conclusion:**

Positive ANA in CSU patients could not indicate the differences in main disease characteristics from the ANA-negative CSU patients. Investigation for ANA may be useful in CSU patients who are suspected of having autoimmune diseases.

## 1. Introduction

Chronic spontaneous urticaria (CSU) is characterized by the presence of wheal and flare with pruritus. Individual wheals last no longer than 24 hours then subside into normal skin, but new rashes can appear elsewhere. Symptoms occur daily or almost daily for at least six weeks. Some patients may also develop swelling of the tissue around the eyes and mouth (angioedema) [1]. CSU is the most common type of chronic urticaria with a prevalence of 0.5-1% of the population [2]. The cause is unknown in most cases, but it is thought to be related to immunological disorders [3]. There are evidences that suggest that autoimmunity may play roles in the etiology of CSU [4–6]. Concomitant autoimmune diseases and CSU are common, for example, autoimmune thyroiditis [4]. A systematic review by Kolkhir et al. [7] showed that some CSU patients can be associated with systemic lupus erythematosus (SLE), especially females [8]. Although CSU does not meet Witebsky's criteria for identifying an autoimmune disorder, there are evidences that a fraction of the CSU population is an autoimmune disease [4, 9].

Antinuclear antibodies (ANA) were discovered in 1957 in the serum of patients with SLE [10, 11]. These antibodies react to antigens in the human nucleus such as Smith, SS-A, SS-B, and double-stranded deoxyribonucleic acid. These antibodies were also later found to be associated with other autoimmune diseases including systemic sclerosis, Sjögren's syndrome, and mixed connective tissue disease (MCTD) [12]. The standard ANA testing method established by the American College of Rheumatology expert panel is the indirect immunofluorescent assay [13]. Other methods are enzyme-linked immunosorbent assay (ELISA) and solid-phase assays (SPA) such as chemiluminescence and fluoroimmunoenzymatic methods that have lower sensitivity but higher specificity [14].

Urticarial rash and urticarial vasculitis are common features in patients with SLE. Up to 22% and 28% of adult patients with SLE present with CSU or CSU-like rash [7]. ANA is often used as a screening test for autoimmune comorbidities in patients with CSU. To our knowledge, there are relatively few studies investigating clinical aspects and comparison between CSU with positive ANA and CSU with negative ANA. Furthermore, most studies were performed by retrospective or cross-sectional study. Only one study was performed by prospective observational study. We, therefore, performed a retrospective cohort study to investigate clinical aspects of patients with CSU who had a positive ANA test and compared them with CSU patients with negative ANA results.

## 2. Methods

This prospective cohort study was approved by the Siriraj Institutional Review Board (SIRB), Faculty of Medicine Siriraj Hospital, Mahidol University, Bangkok, Thailand (SIRB Protocol No. 391/2563 (IRB2), COA No. Si 602/2020).

### 2.1. Patients

From 2013 to 2019, we enrolled patients diagnosed with CSU who visited the Urticaria Clinic at the Department of Dermatology, Siriraj Hospital. CSU was diagnosed by dermatologists based on the patient's history and clinical signs and symptoms. CSU patients aged 18 years or older who gave written informed consent were enrolled. Patients whose medical data were incomplete or could not be traced from medical record files in the Urticaria Clinic were excluded. All patients had at least 18 months of follow-up. Clinical data, demographics, data on laboratory investigations and treatments, and follow-up data were prospectively collected. All patients were investigated for ANA antibodies.

### 2.2. ANA Testing

Immunofluorescent technique with HEp-2 cell substrate was used for ANA testing. The result would be reported as a titer. The cut-off value to define positive ANA was 1 : 100.

### 2.3. Duration of Treatment and Disease

The duration of treatment was defined as the length of time the patient's received treatment until the disease was in remission, or until the last follow-up visit in patients who had not yet recovered. Duration of disease was defined as the time from the onset of urticaria until the disease was cured or until lost to follow up with complete symptom free in spite of no or a very small amount of medication.

### 2.4. Disease Severity Assessment

Urticaria Activity Score summed over 7 days (UAS7) quantifies the daily quantity of wheals (range: 0-none to 3-severe) and daily severity of pruritus (range: 0-none to 3-severe) for seven consecutive days with a maximum weekly score of 42 [15]. UAS7 severity scores are classified as severe urticaria (28–42), moderate urticaria (16–27), and mild urticaria (0–15). UAS7 scores were recorded by each subject during the seven days prior to each visit. The maximal UAS7 scores of all visits were used to represent disease severity.

The medication scoring system proposed by Sussman et al. as a quantitative severity measurement of CSU was also used [16]. The score is calculated based on the sum total of drugs used as follows: antihistamines (2 points, regular dose; 8 points, up to four-fold dose), oral corticosteroids (<11 mg, 5 points; 11–25 mg, 10 points; >25 mg, 15 points), ciclosporin (>3 mg/kg/day, 8 points), hydroxychloroquine (6 points), and leukotriene receptor antagonist (2 points). However, this medication scoring system does not cover omalizumab, which is included in the treatment guideline of urticaria. Therefore, the authors added an omalizumab score as 8 points, which is the same score as ciclosporin. The cumulative medication score was calculated from summation of maximal scores of each medication the patients received since the start of treatment.

### 2.5. Quality of Life Assessment

The Thai versions of the Dermatology Life Quality Index (DLQI) and the Chronic Urticaria Quality of Life Questionnaire (CU-Q2oL), questionnaires validated by Kulthanan et al., were used to assess quality of life [17, 18]. The maximum values of the DLQI and CU-Q2oL for each patient represented their quality of life.

### 2.6. Remission and Relapse

Complete remission was defined as clinical improvement after treatment until achievement of a UAS score of zero without any medication for more than six months. Patients who had a recurrence of CSU after a 6-month period of complete remission were defined to have had a disease relapse.

### 2.7. Statistical Analyses

The demographic data of CSU patients with positive or negative ANA tests were analysed using descriptive analysis. A comparison between CSU patients with positive ANA and negative ANA group was performed using the 2-sample *t*-test and the Mann–Whitney *U* test for quantitative variables with and without normal distribution, respectively. Chi-squared test or Fisher's exact test are used to find the association between categorical data. Chi-squared test was selected and performed when the data in the table is more than 5 in at least 80% of all cells, and no cell contains zero, or else the Fisher's exact test is used. Time to remission and time to relapse were analysed using survival analysis and presented as a Kaplan-Meier curve. Data were analysed using SPSS Statistics version 18.0.

## 3. Results

Three hundred and twenty-three CSU patients were enrolled, and 100 (31%) were positive for ANA (Table 1). The female to male ratio was 5 : 1. There was no significant difference in gender between ANA-positive and ANA-negative groups. The median age of onset in patients with positive ANA was significantly higher than that of patients with negative ANA (38.2 years vs. 35.4 years, *p* = .042). There were no significant differences in disease severity or quality of life between the ANA-positive and ANA-negative groups. The proportion of patients treated with immunosuppressive drugs or omalizumab was higher in the ANA-positive group (32.7% vs. 24.4%, *p* = .128). Patients with ANA had less personal history of atopy than patients without ANA, especially with allergic rhinitis (*p* = .048). There was no significant difference in underlying diseases between the groups. Among ANA-positive patients, there was one case of MCTD, while one case of breast cancer and one case of multiple myeloma were found in ANA-negative CSU patients. Concomitant angioedema was slightly higher in ANA-positive patients (21.0% vs. 18.8%, *p* = .650). Although patients with ANA had a significantly higher levels of ESR (18 vs. 14, *p* = .007), the levels of ESR in both groups were within normal limits (Table 2). No infection was reported in any patients.

**Figure 1:**
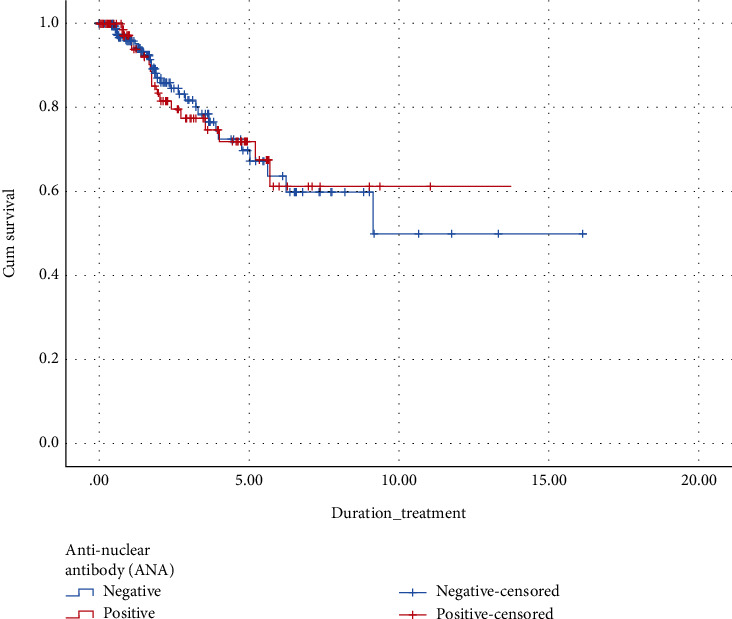
Kaplan-Meier survival curve demonstrating time to remission in patients with CSU with positive antinuclear antibodies (*n* = 100) compared to those with negative antinuclear antibodies results (*n* = 223).

The Kaplan-Meier survival analysis was used to demonstrate “time to remission” and “time to relapse”. People at risk in the study were 323 cases of CSU patients with total time of follow-up of 739.12 person-years. The mean (SD) of time to remission was 10.56 (0.78) years (95% CI: 9.04, 12.08). Forty-six cases (14.2%) achieved complete remission. Approximately 50% of patients did not meet remission status after the follow-up period of at least 10 years (Figure 1). Two percent of ANA-positive CSU patients achieved remission after one year and 28% did so after five years. Four percent of ANA-negative patients achieved remission in one year and 30% did so in five years. There was no statistically significant difference in time to remission (ANA positive: 9.58 years; ANA negative: 10.30 years; *p* = 0.812).

Of the 46 cases with complete remission, there were 17 ANA-positive and 29 ANA-negative cases. One ANA-positive case (5.9%) and six ANA-negative cases (20.7%) had disease relapse (*p* = .234). The mean (SD) time to disease relapse was 2.6 (0.8) years (95% CI 1.1-4.1), and there was no statistically significant difference between both groups (*p* = .251*).* Half of the relapse patients relapsed within 1.3 years.

## 4. Discussion

Many autoimmune diseases were reported in association with CSU patients including SLE, polymyositis, dermatomyositis, and rheumatoid arthritis [19]. CSU tends to affect women more commonly than men [20]. Female CSU patients showed significantly higher incidence of rheumatoid arthritis, Sjögren's syndrome, celiac disease, type I diabetes, and SLE in a large population study than patients without CSU [7]. Male CSU patients showed higher odds of having these autoimmune conditions than controls even though there was no statistical significance [7]. Moreover, CSU patients had been reported to have significantly higher levels of autoantibodies such as ANA, antithyroid peroxidase antibodies, and antimicrosomal antibodies than controls [8]. Vice versa, the reported prevalence of CSU in SLE patients ranges from 0 to 22% [7].

There is a concept of “overlapping autoimmune diseases” which suggests that disorders with autoimmune in nature occur more frequently in patients with known autoimmune diseases [20]. Therefore, clinical relevance and supporting evidence are helpful for physicians to diagnose autoimmune diseases in CSU patients with positive ANA.

Our study revealed a prevalence of ANA positivity of 31%, which was slightly higher than other studies. The prevalence of ANA-positive CSU patients has varied from 10.5% to 29% in other studies [21–26] (Table 3). This may be explained by the different sensitivity and specificity of testing methods and technician experience (14) and ethnic differences in the study populations.

The association between ANA-positive CSU and other underlying diseases remains controversial. It is also unclear about the relevant increased levels of ANAs in CSU patients. Previous studies reported that Sjögren [21, 24], rheumatoid arthritis [21, 24], thyroid autoimmune disease [24], SLE [24], and systemic sclerosis [24] had higher prevalence in ANA-positive CSU. On the contrary, Ertaş et al. [21] reported no significant increase in the prevalence of thyroid disease, diabetes mellitus, hypertension, or asthma in ANA-positive CSU, which was similar to our study. A review of autoimmune comorbidity in CSU [27] reported that the prevalence of overlap syndrome/MCTD was 0.4-0.5%. We found a MCTD prevalence of 0.3% (one case). Magen et al. [24] reported that MCTD has no significant increase in ANA-positive CSU. Although autoimmune disease may contribute to CSU, ANA-positive CSU might not always correlate to the systemic symptoms or other autoimmune diseases [28]. This was consistent with the results of our study. Physiological autoimmunity, which involves a process of elimination of own antigens during cell death, may explain why ANA can be found in healthy people [28]. Thus, in clinical practice, investigation for ANA may be beneficial in CSU patients who are clinically suspected of autoimmune diseases including SLE.

The laboratory characteristics of ANA-positive CSU patients have varied in previous studies. Reported significantly higher positive laboratory tests were anti-TPO [21, 24], antithyroglobulin antibodies [24], total serum IgE [21], basopenia [24], and C-reactive protein [24]. In our study, only ESR level was significantly higher in ANA-positive CSU. Calamita and Pelá Calamita [23] explained that similarities between TPO and self-proteins or exogenous allergens might “confuse the immune system” and trigger an autoimmune reaction. However, we did not find a significant higher positive rate of anti-TPO in ANA-positive CSU patients.

It remains unclear whether disease severity in ANA-positive CSU patients is worse than ANA-negative patients. A study by Magen et al. [24] reported no significant difference assessed by initial UAS and the prevalence of angioedema. In contrast, Ertaş et al. [21] reported a significantly higher prevalence of angioedema. We observed no significant differences, and there were no patients with systemic symptoms in our study.

The pathophysiology of CSU is still not well-understood, but mast cell and basophil activation and degranulation remain central to the process [1]. Once activated, these cells release histamine and other mediators, such as platelet-activating factor and cytokines and wheals and flares result. Antihistamine, the first line treatment, aims to interrupt this mechanism. However, other autoimmune mechanisms may cause an incomplete response to the treatment. Some previous studies suggested that ANA-positive CSU patients tend to be more refractory to treatment [21, 22, 24]. In contrast, we found no significant difference in the cumulative medication score, immunosuppressive drug/omalizumab use, or time to remission in ANA-positive CSU.

As of now, the autoimmune theory is the more widely accepted hypothesis to explain the inappropriate activation of mast cells and basophils. Two endotypes of CSU have been described [20, 29]. Type I autoimmune CSU (aiCSU), also called autoallergic CSU, is mediated by IgE autoantibodies, and Type IIb aiCSU is mediated by antibodies against IgE and its high affinity receptor, Fc*ε*RI. Type IIb aiCSU was reported to have high disease activity, high rates of autoimmune comorbidity, and poor response to treatment with antihistamines and omalizumab. Tests for Type IIb aiCSU included autologous serum skin test (ASST), autoantibody immunoassays, and basophil activation testing. However, these tests are not widely available and have limitations, i.e., some are invasive, not sufficiently validated, and/or cost intensive.

The International EAACI/GA^2^LEN/EuroGuiDerm/APAAACI Guideline for the definition, classification, diagnosis, and management of urticaria recommends the aims of diagnostic workup in CSU including to confirm the diagnosis and exclude differential diagnoses, to look for the underlying causes, to identify relevant conditions that modify disease activity, to check for comorbidities, to identify the consequences of CSU, to assess predictors of the course of disease and response to treatment; and to monitor disease activity, impact, and control [30]. The basic tests include a differential blood count and CRP and/or ESR, in all patients. Further diagnostic testing may be performed as indicated.

We suggested that for ANA laboratory investigation, besides aiming for underlying SLE or other autoimmune diseases in suspected cases, positive ANA test in CSU patients may suggest autoimmune in nature. Further studies are needed to clarify.

## 5. Conclusions

We conducted a retrospective cohort study of the clinical features and the course of disease of CSU patients with positive ANA results and compared them with ANA-negative patients. We found no significant differences in disease symptoms, severity, associated diseases, laboratory results, disease duration, and treatment outcomes. However, CSU are reported in association with many autoimmune diseases. Thus, we suggest that investigation for ANA may be useful in CSU patients who have clinically suspected autoimmune disease.

## Figures and Tables

**Table 1:** Clinical features of patients with chronic spontaneous urticaria (CSU) with positive or negative antinuclear antibodies (ANA).

	Patients with CSU (%)		*p* value
	Positive ANA (*n* = 100)	Negative ANA (*n* = 223)	
Sex, *n* (%)			0.848
Male	17/100 (17.0)	36/223 (16.1)	
Female	83/100 (83.0)	187/223 (83.9)	
Age of onset (year), median (min-max)	38.2 (8.4-81.4)	35.4 (7.2-74.9)	0.042^∗^
Age of patients' first visit (year), median (min-max)	39.2 (12.9-82.1)	36.9 (15.0-75.9)	0.033^∗^
Duration of treatment (year), median (min-max)	1.73 (0.01-13.74)	1.21 (0.00-16.15)	0.101
Duration of disease (year), median (min-max)	2.73 (0.04-23.49)	2.38 (0.15-41.19)	0.164
Disease severity			
Maximal UAS7, median (min-max)	18 (1-42)	16 (2-42)	0.591
Mild, *n* (%)	25/53 (47.2)	56/117 (47.9)	
Moderate, *n* (%)	16/53 (30.2)	34/117 (29.1)	
Severe, *n* (%)	12/53 (22.6)	27/117 (23.1)	
Maximal CU-Q2oL, median (min-max)	25 (0-83)	24 (1-82)	0.873
Maximal DLQI, median (min-max)	5.5 (0-27)	4 (0-25)	0.730
Cumulative medication score, median (min-max)	8 (2-34)	8 (2-29)	0.152
Mild, *n* (%)	9/98 (9.2)	23/217 (10.6)	0.248
Moderate, *n* (%)	66/98 (67.3)	160/217 (73.7)	
Severe, *n* (%)	23/98 (23.5)	34/217 (15.7)	
Immunosuppressive drug/biologic drug, *n* (%)	32/98 (32.7)	53/217 (24.4)	0.128
Personal history of atopy, *n* (%)	23/100 (23.0)	68/223 (30.5)	0.166
Allergic rhinitis, *n* (%)	13/100 (13.0)	50/223 (22.4)	0.048^∗^
Asthma, *n* (%)	6/100 (6.0)	7/223 (3.1)	0.233
Atopic dermatitis, *n* (%)	1/100 (1.0)	4/223 (1.8)	1.000
Allergic conjunctivitis, *n* (%)	1/100 (1.0)	7/223 (3.1)	0.443
Family history of atopy, *n* (%)	29/100 (29.0)	68/223 (30.5)	0.787
Allergic rhinitis, *n* (%)	10/100 (10.0)	32/223 (14.3)	0.283
Asthma, *n* (%)	7/100 (7.0)	13/223 (5.8)	0.687
Atopic dermatitis, *n* (%)	0/100 (0.0)	3/223 (1.3)	0.555
Allergic conjunctivitis, *n* (%)	0	0	—
Urticaria/angioedema, *n* (%)	2/100 (2.0)	5/223 (2.2)	1.000
Underlying diseases			
Thyroid disease, *n* (%)	8/100 (8.0)	12/223 (5.4)	0.367
Diabetes mellitus, *n* (%)	4/100 (4.0)	9/223 (4.0)	1.000
Hyperlipidemia, *n* (%)	2/100 (2.0)	10/223 (4.5)	0.355
Hypertension, *n* (%)	7/100 (7.0)	12/223 (5.4)	0.568
Associated angioedema, *n* (%)	21/100 (21.0)	42/223 (18.8)	0.650

^∗^ indicated statistical significance.

**Table 2:** Laboratory investigations of patients with chronic spontaneous urticaria (CSU) with positive and negative antinuclear antibodies (ANA).

	Patients with CSU		*p* value
	Positive ANA (*n* = 100)	Negative ANA (*n* = 223)	
Total eosinophil (cell/microL), median (min-max)	113 (13-399)	148 (11-746)	0.177
Eosinophilia (≥500 eosinophils/microL), *n* (%)	3/71 (4.2%)	9/148 (6.1%)	0.755
Total white blood cell (cell/microL), median (min-max)	6,380 (2,053-13,100)	6,385 (2,048-17,220)	0.748
Leukopenia (<4,000 white blood cell/microL), *n* (%)	22/94 (23.4%)	58/206 (28.2%)	0.388
Erythrocyte sedimentation rate (ESR) (mm/hr) (normal ≤ 20 mm/hr), median (min-max)	18 (4-84)	14 (1-60)	0.007^∗^
Stool exam: Blastocystis hominis, *n* (%)	1/42 (2.4%)	4/85 (4.7%)	1.00
HBsAg (positive), *n* (%)	1/57 (1.8%)	5/127 (3.9%)	0.668
Anti HCV (positive), *n* (%)	2/52 (3.8%)	0/104 (0.0%)	0.110
Thyroid microsomal antibody (positive), *n* (%)	1/15 (6.7%)	3/30 (10.0%)	1.000
Antithyroid peroxidase (anti-TPO) (positive), *n* (%)	8/40 (20.0%)	11/75 (14.7%)	0.463
Antithyroglobulin antibody (positive), *n* (%)	15/52 (28.8%)	22/109 (20.2%)	0.222
T3, *n* (%)			0.713
Low	2/19 (10.5%)	7/45 (15.6%)	
FT4, *n* (%)			0.614
Low	3/24 (12.5%)	2/42 (4.8%)	
High	1/24 (4.2%)	2/42 (4.8%)	
TSH, *n* (%)			0.834
Low	1/58 (1.7%)	5/122 (4.1%)	
High	3/58 (5.2%)	7/122 (5.7%)	
D-dimer (>500 ng/mL), *n* (%)	7/14 (50.0%)	14/37 (37.8%)	0.431
IgE (>100 IU/mL), *n* (%)	13/18 (72.2%)	22/38 (57.9%)	0.301
Autologous serum skin test (positive), n (%)	4/18 (22.2%)	14/39 (35.9%)	0.302
Skin prick pest (positive), *n* (%)	6/15 (40.0%)	26/45 (57.8%)	0.232

^∗^ indicated statistical significance.

**Table 3:** Previous studies of chronic spontaneous urticaria with antinuclear antibodies positive results compared to our study.

Authors, year study design	Study population			ANA method	Prevalence of positive ANA	Comparing between ANA-positive CSU patients and ANA-negative CSU patients				Others
	*N*	F : M ratio	Country			Disease severity	Associated disease	Laboratory characteristics	Treatment outcome	
Viswanathan et al., [22] Retrospective study	195 CSU patients	2.8 : 1	USA	N/A	29%	N/A	N/A	N/A	Significant higher prevalence of patients refractory to antihistamines with or without the use of a LTRA (50% vs. 30%), *p* = 0.04	Positive ANA has a significant odds ratio for identifying patients refractory to antihistamines with or without the use of a LTRA (OR 2.3), *p* = 0.04
Calamita et al., [23] Cross-sectional study	67 CSU patients	2.2 : 1	Brazil	IIF using HEp-2 cells	10.5%	N/A	N/A	N/A	N/A	Significant association between positive ANA and presence of anti-TPO (OR 5.94)
Magen et al., [24] Retrospective study	-91 ANA-positive CSU patients -478 ANA-negative CSU patients -3131 ANA positive non-CSU patients	3.6 : 1	Israel	Fully automated Luminex-based system (BioPlex 2200 ANA screen, by Bio-Rad; Bio-Rad Laboratories, Hercules, CA)	16%	No significant difference - initial UAS (3.8 ±0.9 VS 3.7 ± 0.7), p =0.235 - prevalence of angioedema (29.7% VS 23.8%), *p* = 0.235	Significant difference - Sjögren's syndrome (3.3% vs. 0%), *p* = 0.004 - graves' disease (5.5% vs. 1.7%), *p* = 0.042 - Hashimoto's thyroiditis (23.1% vs. 14.2%), *p* = 0.040 - SLE (4.4% vs. 0%), *p* = <0.001 - systemic sclerosis (2.2% VS 0%), p =0.025 - overlap syndrome (3.3% VS 0%), p =0.004 No significant difference - rheumatoid arthritis (2.2% VS 0.6%), p =0.182 - mixed connective tissue disease (0% VS 0%), p = 1	Significant difference - anti-TPO (27.5% vs. 12.2%), *p* = <0.001 - Antithyroglobulin antibody (20.9% vs. 10.4%), *p* = <0.001 - Basopenia (0.04 ± 0.09 VS 0.15 ± 0.11), p = <0.001 - C-reactive protein (6.4 ± 10.3 VS 4.1 ± 8.8), p = 0.027 No significant difference - IgE (U/mL) (185.5 ± 346.6 VS 126.4 ± 309.8), p = 0.102	Significant higher prevalence of patients resistant to four-fold standard licensed doses of antihistamines (12.1% VS 6.1%), p =0.046	
Ye et al., [25] Prospective observational study	75 CSU patients	2.1 : 1	South Korea	IIF using HEp-2 cells	10.7%	N/A	N/A	N/A	N/A	
Campos et al., [26] Cross-sectional case-control	- 27 CSU patient - 22 controls (other hypersensitivities without urticaria)	2.4 : 1	Brazil	Immunoassay	14.8%	N/A	N/A	N/A	N/A	
Ertaş et al., 2020 [21] Retrospective study	447 CSU patients	2.4 : 1	Turkey	IIF using HEp-2 cells	23.9%	Significant higher prevalence of angioedema (70% vs. 53%), *p* = 0.002	Significant difference - Sjögren's syndrome (4.7% vs. 0.6%), *p* = 0.003 - rheumatoid arthritis (10.3% vs. 4.4%), *p* = 0.025 No significant difference - thyroid disease (24% **vs.** 18%), *p* = 0.128 - diabetes mellitus (24% VS 18%), p = 0.128 - hypertension (9% VS 11%), p = 0.651 - asthma (20% VS 17%), p = 0.552	Significant difference - anti-TPO (27% VS 18%), *p* = 0.049 - Total IgE (64 (21-160) vs. 110 (44-236)), *p* = 0.001 No significant difference - ESR (11 (5-18) Basopenia 8 (4-16)), *p* = 0.128 - CRP (3.3 (3.0-7.5) vs. 3.3 (3.0-6.8)), p = 0.765	Significant higher prevalence of nonresponders (VAS improvement< 20% at week 12 of 300 mg/4weeks omalizumab treatment (45% vs. 9%), p < 0.001	
Our study Prospective study	323 CSU patients	5.1 : 1	Thailand	IIF using HEp-20-10 cells	31%	No significant difference - maximal UAS7 (17 (0-42) vs. 15 (0-42)), *p* = 0.851 - prevalence of angioedema groups (21.0% vs. 18.8%), *p* = 0.650	No significant difference - thyroid disease (8.0% vs. 5.4%), *p* = 0.367 - diabetes mellitus (4.0% vs. 4.0%), *p* = 1.000 - hyperlipidemia (2.0% vs. 4.5%), *p* = 0.355 - hypertension (7.0% vs. 5.4%), p = 0.568	Significant difference - ESR (18.00 (4-84) vs. 14.00 (1-60)), *p* = 0.007 No significant difference - anti-TPO (20.0% vs. 14.7%), *p* = 0.463	No significant difference - cumulative medication score (8 (2-34) vs. 8 (2-29)), *p* = 0.152 - immunosuppressive drug/omalizumab use (32.7% vs. 24.4%), *p* = 0.128 - time to remission (9.6 years vs. 10.3 years)	

Abbreviations: ANA: antinuclear antibodies; anti-TPO, and anti-thyroid peroxidase; CSU: chronic spontaneous urticaria; F: female; LTRA: leukotriene receptor antagonist; M: male; MCTD: mixed connective tissue disease; N/A: not available; SLE: systemic lupus erythematosus.

## Data Availability

The data used to support the findings of this study are available from the corresponding author upon request.
